# A prediction model for hypoxemia during routine sedation for gastrointestinal endoscopy

**DOI:** 10.6061/clinics/2018/e513

**Published:** 2018-11-06

**Authors:** Wujun Geng, Danyu Jia, Yichuan Wang, Shenhui Jin, Yelong Ren, Dongdong Liang, Aote Zheng, Hongli Tang, Zarrin Basharat, Vincent Zimmer, Simon Stock, Maddalena Zippi, Wandong Hong

**Affiliations:** I Department of Anesthesiology, the First Affiliated Hospital of Wenzhou Medical University, Wenzhou, Zhejiang, People's Republic of China; II Microbiology & Biotechnology Research Lab, Department of Environmental Sciences, Fatima Jinnah Women University, 46000 Rawalpindi, Pakistan; III Department of Medicine II, Saarland University Medical Center, Saarland University, Homburg, Germany; IV Department of Medicine, Marienhausklinik St. Josef Kohlhof, Neunkirchen, Germany; V Department of Surgery, World Mate Emergency Hospital, Battambang, Cambodia; VI Unit of Gastroenterology and Digestive Endoscopy, Sandro Pertini Hospital, Rome, Italy; VII Department of Gastroenterology and Hepatology, the First Affiliated Hospital of Wenzhou Medical University, Wenzhou, Zhejiang, People's Republic of China

**Keywords:** Risk Factor, Age, Snoring, Hypoxemia, Endoscopic Sedation, Anesthesia

## Abstract

**OBJECTIVES:**

The current study was designed to assess the clinical predictors of hypoxemia and to develop a multivariable, predictive model for hypoxemia during routine gastrointestinal endoscopy.

**METHODS:**

In total, 308 patients were enrolled in the analysis. Demographic data, concurrent chronic disease information, anesthetic dose and Modified Observer's Assessment of Alertness/Sedation (MOAA/S) scores were collected and analyzed statistically.

**RESULTS:**

Multivariate logistic regression indicated that age (OR: 1.04; 95%CI 1.01-1.08), body mass index (BMI) (OR: 1.12; 95%CI: 1.02-1.21) and habitual snoring (OR: 3.71; 95%CI: 1.62-8.48) were independently associated with hypoxemia. A logistic regression function (LR model) was developed to predict hypoxemia considering the parameters of -7.73+0.04 age (years), +0.11 BMI, and +1.31 habitual snoring (yes or no). The area under the receiver operating characteristic (ROC) curve for the LR model was 0.76.

**CONCLUSIONS:**

The LR model, consisting of age, BMI and habitual snoring, was a useful predictor of hypoxemia during routine sedation for gastrointestinal endoscopy.

## INTRODUCTION

Drug-induced sedation in endoscopic procedures improves patient comfort and facilitates endoscopic performance 1. Propofol-mediated sedation has become increasingly popular during endoscopic procedures because of its rapid onset of action and short duration of effect 2. Propofol may give rise to sedation-related complications by accentuating airway collapse and lowering the threshold for upper airway obstruction.

Numerous studies have focused on identifying risk factors for sedation-related complications that occur during endoscopic procedures. Mehta et al. 3 found that increased patient age, higher loading propofol dose, and smoking were all associated with higher rates of sedation-related adverse events. Cote et al. 2 reported that patients with obstructive sleep apnea (OSA) are thought to have a greater risk for developing sedation-related complications during advanced endoscopic procedures.

Among propofol sedation-related complications, the incidence of hypoxemia, hypotension and premature procedure termination during advanced endoscopic procedures were 12.8%, 0.5% and 0.6%, respectively 4. Hence, hypoxemia is the most common sedation-related complication during advanced endoscopy. Though it is usually transient and mild, and spontaneous recovery is likely, hypoxemia remains the dominant cause of increased morbidity and mortality 5. It may result in lethal hypoxemia requiring emergency airway management and risks interrupting or ending the endoscopic procedure. Some of the approaches that are helpful in alleviating hypoxemia include increasing supplemental oxygen, inserting a nasopharyngeal airway, performing various airway maneuvers such as chin lifting, providing positive pressure ventilation, and achieving an open airway via endotracheal intubation 1,5. Early identification of high-risk patients prior to endoscopy may help the physician select patients who would benefit most from an aggressive intervention. Thus, a reliable method of risk stratification for hypoxemia during endoscopic procedures is of significant clinical importance.

In the literature, there is limited information on the specific clinical predictors of developing hypoxemia during routine gastrointestinal endoscopy. The current study, therefore, was aimed at assessing the clinical predictors of hypoxemia and subsequently developing a multivariable model for predicting hypoxemia during routine gastroenterology procedures.

## MATERIALS AND METHODS

### Inclusion and exclusion criteria

We performed a prospective cohort study of patients undergoing routine esophagogastroduodenoscopy (EGD) and/or colonoscopy in the Department of Gastroenterology and Hepatology at the First Affiliated Hospital of Wenzhou Medical University between July 1, 2017, and July 31, 2017. The exclusion criteria included therapeutic endoscopy, prior gastric or colonic resection, inadequate bowel preparation, severe cardiopulmonary diseases such as myocardial infarction, bronchial asthma, organ failure preceding data collection, American Society of Anesthesiologists (ASA) classification of 4 or higher and a lack of complete data availability 6.

### Patient monitoring and data collection

During the examination prior to endoscopy, each patient's status was classified according to the physical status classification of the ASA 7,8. For induction, the anesthesiologist used propofol alone or in combination with low-dose etomidate. The propofol dosage was adjusted to maintain deep sedation throughout the procedure. The depth of sedation was assessed by the anesthesiologist using the Modified Observer's Assessment of Alertness/Sedation (MOAA/S) score at the time of the endoscopic examination 2. All patients received oxygen via a nasal cannula at 3 L/min and underwent continuous electrocardiography. Heart rate, pulse oximetry and blood pressure (intermittently) were monitored during the procedure.

Age, gender, BMI, ASA classification, history of hypertension, history of diabetes mellitus, alcohol consumption, smoking history, and history of habitual snoring (yes or no) were recorded prior to endoscopy. The specific endoscopic procedure, doses of propofol and etomidate and MOAA/S score were collected during and after the procedure. The subjects were classified as alcohol consumers if they had consumed any alcoholic beverage at least once per week during the preceding 6 months 9. Subjects were classified as cigarette smokers if they had smoked 10 or more cigarettes per week during the preceding 6 months 9. Habitual snoring was defined as snoring for more than 3 nights per week 10.

### Definition of outcome and ethics

The primary endpoint of this study was to develop a model to predict hypoxemia during endoscopic examination. Hypoxemia was defined as a pulse oximetry <90% for any duration during the endoscopic procedure 4.

This study protocol was approved by the Ethics Committee of the First Affiliated Hospital of Wenzhou Medical University and performed according to the principles articulated in the Declaration of Helsinki. Informed consent was obtained from all subjects, and the data were anonymized before analysis.

### Statistical analysis

Continuous values are expressed as the means±SD and were compared using Student's t-tests. Categorical values are described by counts and proportions and were compared by the χ^2^ test or Fisher's exact test.

All the variables determined to be different between patients with and without hypoxemia through univariate analysis were included as eligible factors in a forward-conditional stepwise LR analysis. For this analysis, the conditional probabilities for stepwise entry and removal of a factor were 0.05 and 0.10, respectively 11,12. Based on the results of multiple logistic regression analysis, a logistic regression equation model was developed to predict hypoxemia. Model calibration, reflecting the link between the predicted and observed risks, was determined by the Hosmer-Lemeshow goodness of fit test 13. Odds ratios (ORs) were calculated with 95% confidence intervals (CIs).

The area under the ROC curve (AUC) was used to evaluate the performance of the predictions. A variable with an AUC above 0.7 was considered useful 11.

The sensitivity, specificity, positive likelihood ratio (+LR), negative likelihood ratio (-LR), positive predictive value, negative predictive value, and diagnostic accuracy were calculated for the cutoff value. The best Youden Index (sensitivity + specificity − 1) value was used to determine the best cutoff point of the logistic model to predict hypoxemia 14.

Differences were considered statistically significant if the two-tailed *p*-value was less than 0.05.

## RESULTS

### Baseline characteristics

During the study period, 308 patients with a median age of 50±12.0 years were enrolled. One hundred seventy-eight (58.1%) were male, and the remainder were female. In all, 36 patients (11.7%) underwent only EGD, while 55 (17.9%) received only colonoscopies. The number of patients subjected to a combination of EGD and colonoscopy examinations was 217 (70.4%). Hypoxemia occurred in 29 patients (9.4%). There were no cases of endotracheal intubation in the entire cohort.

### Univariable and multivariable analysis

As shown in Table 1, the univariate analysis indicated that age, BMI, habitual snoring and MOAA/S score were significantly associated with hypoxemia. Patients with hypoxemia were older and had a higher mean BMI than those without hypoxemia. Additionally, the proportion of patients with habitual snoring was higher among patients who experienced hypoxemia than among those who did not experience hypoxemia. Furthermore, the mean MOAA/S score of patients with hypoxemia was higher than that of patients without hypoxemia. This result means that patients with hypoxemia were more lightly sedated than patients without hypoxemia. There were no statistically significant differences between patients with and without hypoxemia with respect to sex, ASA classification, hypertension, diabetes mellitus, alcohol consumption, smoking, propofol dosage, type of endoscopic examination, dose of induction or proportion of etomidate use.

A multivariate analysis was performed based on LR for age, BMI, habitual snoring and MOAA/S score. Age (OR: 1.04; 95%CI: 1.01-1.08; *p*=0.024), BMI (OR: 1.12; 95%CI: 1.02-1.21; *p*=0.009) and habitual snoring (OR: 3.71; 95%CI: 1.62-8.48; p=0.002) were independently associated with hypoxemia.

### Model development, calibration and performance

An LR model was developed that was aimed at predicting hypoxemia, with input values of -7.73+0.04 age (years), +0.11 BMI, and +1.31 habitual snoring (yes or no). The Hosmer-Lemeshow goodness of fit test did not reach statistical significance (*p*=0.185), suggesting that our prediction model fit the observed data well.

**Figure 1:**
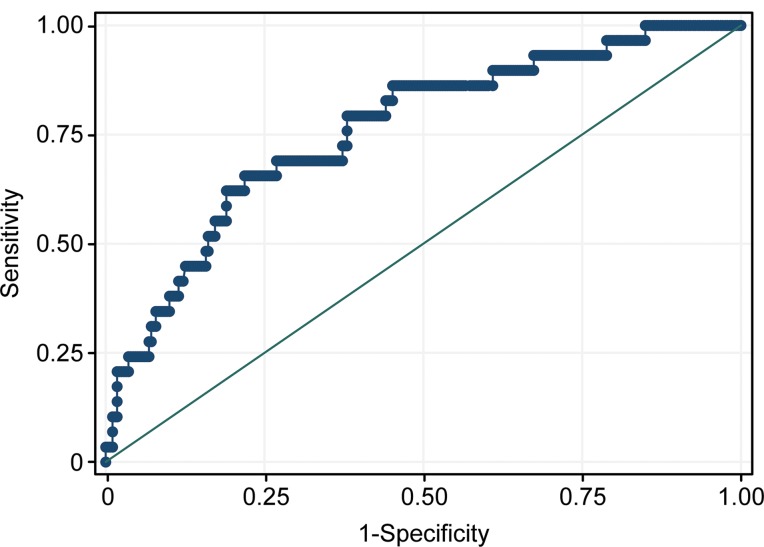
ROC curve for the implemented LR model to predict hypoxemia. The AUC for the LR model developed for the prediction of hypoxemia was 0.76±0.05. The ideal AUC was 1.00. The reference line represents an AUC of 0.50, which is based on chance alone.

As shown in Figure 1, the AUC for the LR model for the prediction of hypoxemia was 0.76±0.05, which means that the LR model was a useful predictor of hypoxemia, with an AUC of more than 0.7.

Based on the ROC curve analysis, the optimum cutoff value for the LR model was -1.96. The sensitivity, specificity, +LR, -LR, positive predictive value, negative predictive value, and diagnostic accuracy were 66%, 78%, 3.00, 0.44, 24%, 96% and 77% respectively.

**Figure 2:**
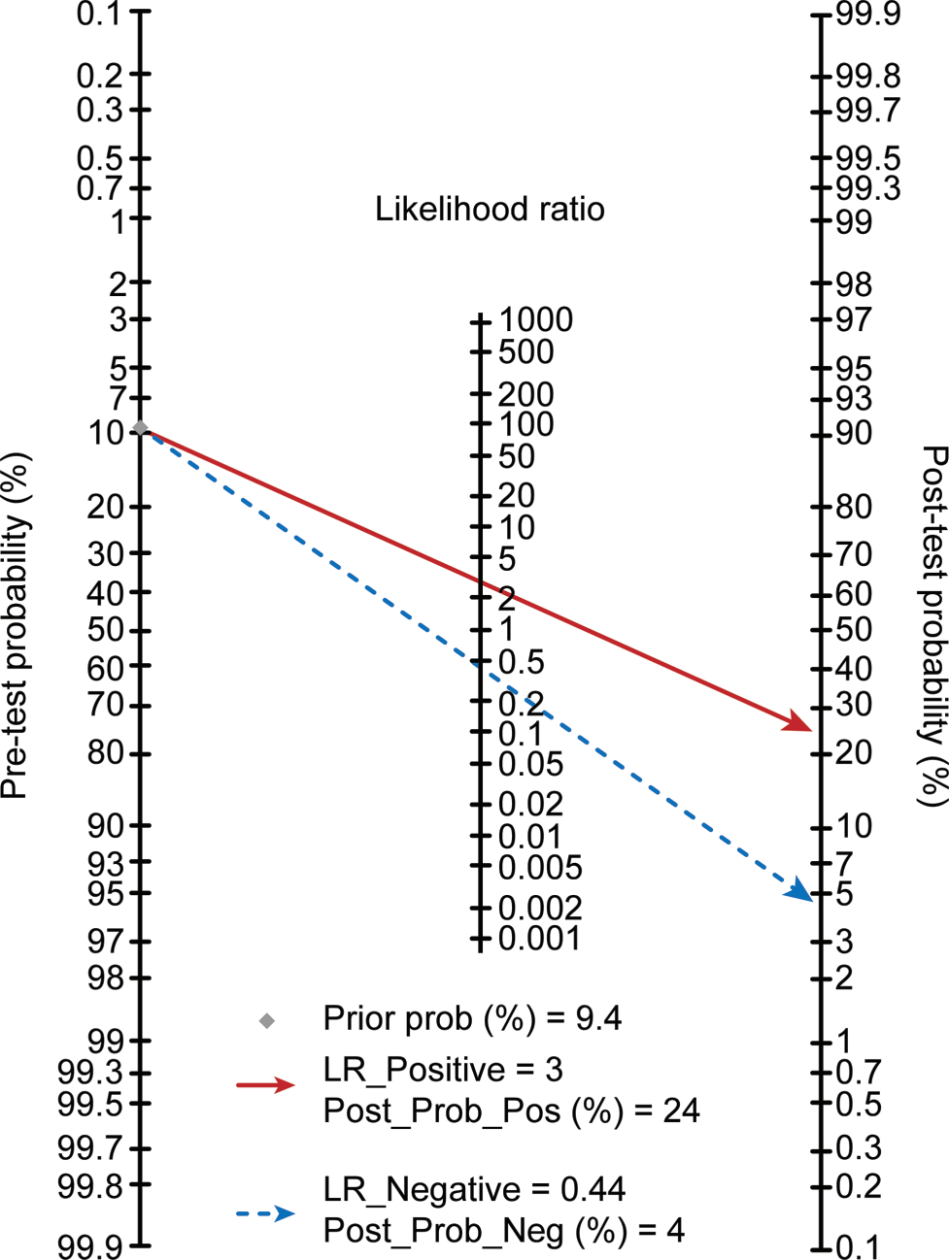
Fagan plot of the LR model that was used for the prediction of hypoxemia.

Using a cutoff value of -1.96 and the prevalence value of hypoxemia (9.4% in this study) as the pretest probability, the Fagan plot (Figure 2) indicated that this LR model can be clinically informative, as it increases the probability up to 24% of being classified as hypoxemic when positive and lowers the probability to 4% when negative.

## DISCUSSION

The results of the this study demonstrated the following: (i) the incidence of hypoxemia was 9.4%, and age (OR 1.04; 95%CI: 1.01-1.08), BMI (OR 1.12; 95%CI: 1.02-1.21) and habitual snoring (OR 3.71; 95%CI: 1.62-8.48) were independently associated with hypoxemia and (ii) the LR model, consisting of age, BMI and habitual snoring, was a useful predictor of hypoxemia, with an AUC greater than 0.7 (AUC=0.76±0.05) (Figure 1).

Compared to young healthy adults, healthy older subjects had reduced diaphragmatic strength. This means that age-related muscular atrophy is a factor in decreasing fast twitch muscle fibers within the diaphragm 15,16. This may predispose older individuals to diaphragmatic fatigue and ventilatory failure during periods of increased ventilatory load on the respiratory system 16. In addition, older subjects have a reduced ventilatory response to hypoxia and hypercapnia. Potentially, this can cause air trapping and hyperinflation secondary to decreased chest wall compliance and higher residual lung volume. Older patients have increased pulmonary airspace when compared to young healthy adults 16,17. Mehta et al. 3 reported that greater patient age was associated with higher rates of sedation-related adverse events. As expected, our study revealed that age (OR: 1.04; 95%CI 1.01-1.08) was independently associated with hypoxemia during endoscopic procedures.

Obesity has long been recognized as having significant effects on respiratory function 18. Jones et al. 19 reported that lung volumes, especially functional residual capacity and expiratory reserve volume, decrease as BMI increases. Obese patients tend to have higher respiratory rates, lower tidal volumes and increased airway resistance 18. Littleton et al. 20 assessed the association between obesity and hypoxemia in patients with no apparent cardiac or pulmonary disease and demonstrated that as the BMI increased, the arterial partial pressure of oxygen decreased, and the alveolar-arterial gradient increased. Kendale et al. 21 proposed that a high BMI was associated with the occurrence, severity, and prolonged duration of hypoxemia in noncardiac surgery. Wani et al. 22 suggested that obesity is a risk factor for sedation-related complications during propofol-mediated sedation for advanced endoscopic procedures. As expected, our study showed that BMI (OR: 1.12; 95%CI: 1.02-1.21) was independently associated with hypoxemia.

The relationship between the snoring, tiredness, observed apnea, blood pressure, body mass index, age, neck circumference and gender (STOP-BANG) (a screening tool for OSA) score and sedation-related complications that arise during endoscopic procedures has been investigated in the literature. Cote et al. 2 reported that patients with a high risk of OSA (as identified by STOP-BANG) are thought to have a greater risk for developing sedation-related complications during advanced endoscopic procedures. Snoring is one of the major symptoms of obstructive sleep apnea-hypopnea syndrome. Habitual snoring (i.e., snoring for more than 3 nights per week) has been reported as the best predictor of OSA 10. As expected, our study showed that habitual snoring (OR: 3.71; 95%CI 1.62-8.48) was independently associated with hypoxemia.

There is no consensus in the literature regarding the risk factors for sedation-related adverse events during gastrointestinal endoscopy. Mehta et al. suggested that age, higher loading propofol doses, and smoking were associated with higher rates of sedation-related adverse events during EGD or colonoscopy 3. Cote et al. noted that BMI, male sex, and an ASA classification of 3 or higher but not propofol dose were independent predictors of airway management modifications during advanced endoscopic procedures 4. Our data showed that age, BMI and habitual snoring, but not total propofol dosage, induction dose, proportion of etomidate use, sex, ASA classification, hypertension, diabetes mellitus, alcohol consumption, smoking or type of endoscopic examination were associated with hypoxemia. One possible explanation for these differences among studies might be the variations among study populations regarding the proportion of patients with advanced ASA classifications, smoking, etc. The proportion of smokers and the proportion of patients with an ASA classification of 3 or higher in our data were 22.7% (70/308) and 0.6% (2/308), respectively, whereas the same proportions were 54.0% (142/263) and 61.2% (161/263), respectively, in the study by Mehta et al. 3.

As expected, the LR model, consisting of the abovementioned three parameters and a cutoff value of -1.96, achieved an acceptable sensitivity of 66% and specificity of 78%. The application of the proposed LR model is expected to change current clinical practice in routine gastrointestinal endoscopy examinations involving sedation. As shown in the Fagan plot (Figure 1), if the LR model value was greater than or equal to -1.96 in a patient, the probability of developing hypoxemia during endoscopy increased from 9.4% to 24%, and if the LR model value was less than -1.96, the probability decreased to 4%. This indicates that patients with an LR model value less than -1.96 will have a 96% chance of avoiding hypoxemia. Therefore, this subgroup of patients could be considered low risk for hypoxemia during endoscopy with sedation. Patients with high LR model values or with old age, obesity and habitual snoring should be labeled high risk for hypoxemia. Therefore, to decrease the likelihood or to prevent the incidence of hypoxemia, continuous monitoring of breathing in patients who are considered high risk for hypoxemia is necessary 5. In addition, increasing the concentration of inspired oxygen and performing chin lift maneuvers and jaw thrusts are simple, effective strategies to prevent hypoxemia. Furthermore, routine use of the nasopharyngeal airway during general anesthesia in this subgroup of patients might reduce the frequency of hypoxemic events during endoscopic sedation 1. Ultimately, Cohen suggested that an ideal short-acting sedative agent such as propofol, without adjunctive agents such as opioids or benzodiazepines, would be most appropriate for high-risk patients 23.

The strengths of our study include the following: (i) novelty: to the best of our knowledge, this is the first prospective study to investigate the risk factors of hypoxemia during sedation for routine gastrointestinal endoscopy; (ii) concurrent EGD and colonoscopy association analysis: most (217/318, 70.5%) of our patients underwent EGD and colonoscopy simultaneously, while previous studies reported patients undergoing either EGD or colonoscopy alone 3,4, making our results more significant and more applicable to the Chinese people and the clinical conditions in China when compared to previous studies 3,4; and (iii) while it is well known that a higher loading propofol dose is associated with higher rates of sedation-related adverse events, there was no statistically significant difference between patients with and without hypoxemia with respect to induction dose or total propofol administration in conjunction with etomidate use (Table 1). Moreover, the mean MOAA/S scores of patients with hypoxemia were higher than those of patients without hypoxemia. This means that patients with hypoxemia were more lightly sedated than patients without hypoxemia. Therefore, the incidence of hypoxemia in our results may be less biased due to propofol usage. Our study also had several important limitations. First, the sample size of the study was relatively small. Second, the performance of our LR model was not validated on an external data set. The authors want to clearly state that an external validation is mandatory before it is used in clinical practice. In addition, the endoscopic procedure time was not evaluated as a potential risk factor for hypoxemia since this was not imperative to complete the study. A literature search demonstrated that endoscopic procedure time was not associated with the incidence of hypoxemia during endoscopy with sedation 3,4,22.

In conclusion, age, BMI and habitual snoring were independently associated with hypoxemia, and the LR model, consisting of age, BMI and habitual snoring, was a useful predictor of hypoxemia during sedation for routine gastroenterology procedures.

## AUTHOR CONTRIBUTIONS

All authors contributed toward analyzing the data and drafting and revising the paper and agree to be accountable for all aspects of the work herein. All the authors have read and approved the final manuscript.

## Figures and Tables

**Table 1:** Baseline characteristics and clinical outcomes of 308 patients in the two study groups.

Variable	Hypoxemia N=29	No-hypoxemia N=279	*p*-value
Age, years	55.5±11.3	49.5±12.0	0.01
Male sex, N (%)	21 (72.4)	158 (56.6)	0.101
BMI, kg/m^2^	25.3±3.8	22.9±4.0	<0.001
ASA class I/II/III	4/25/0	97/180/2	0.05
Hypertension, N (%)	9 (31.0)	47 (16.9)	0.059
Diabetes mellitus, N (%)	2 (6.9)	22 (7.9)	0.85
Alcohol consumption, N (%)	10 (34.5)	88 (31.5)	0.746
Smoking, N (%)	7 (24.1)	63 (22.6)	0.849
Habitual snoring, N (%)	19 (65.5)	83 (29.8)	<0.001
Endoscopy			0.903
Gastroscopy only, N (%)	3 (10.3)	33 (11.8)	
Colonoscopy only, N (%)	6 (20.7)	49 (17.7)	
Gastroscopy and colonoscopy, N (%)	20 (69.0)	197 (70.5)	
Pharmacologic data			
Induction propofol dose	120±35	125.9±26	0.360
Total propofol dose	182±53	169±45	0.133
Etomidate, N (%)	7 (24.1)	61 (21.9)	0.779
MOAA/S score during the procedure	1.7±0.7	1.5±0.6	0.019

(a) Data are shown as means±standard deviation or numbers and percentages, as appropriate. N=number.
